# Supplementary material for: Biodegradation of the endocrine-disrupting compound bisphenol F by Sphingobium yanoikuyae DN12
Source: Appl Environ Microbiol. 2025 Nov 6;91(12):e01830-25. doi: 10.1128/aem.01830-25 (PMC12724174; doi:10.1128/aem.01830-25)
Supplement: Supplemental material — Figures S1 to S18; Tables S1 to S4. [file aem.01830-25-s0001.pdf]

Supplemental Material

Biodegradation of the endocrine-disrupting compound bisphenol F by *Sphingobium yanoikuyae* DN12

Ruomu Chen [1], Yutian Gan [1], Wanting Huang [1], Chenyang Wang [1], Junhong Ge [1], Yuanyuan Cheng [1], Wenjing Qiao [1], Jiandong Jiang [1,*], Kai Chen [1,*]

[1] Department of Microbiology, College of Life Sciences, Nanjing Agricultural University, Key Laboratory of Agricultural and Environmental Microbiology, Ministry of Agriculture and Rural Affairs, Nanjing 210095, China

***Corresponding Author:**

Kai Chen

E-mail: chenkai@njau.edu.cn; Tel: 86-25-84399726

Jiandong Jiang

E-mail: jiang_jjd@njau.edu.cn; Tel: 86-25-84399726

Running title: Bacterial Catabolism of Bisphenol F

Key words: Bisphenol F, biodegradation, two-component oxidoreductase, Baeyer-Villiger monooxygenase, *Sphingobium yanoikuyae*

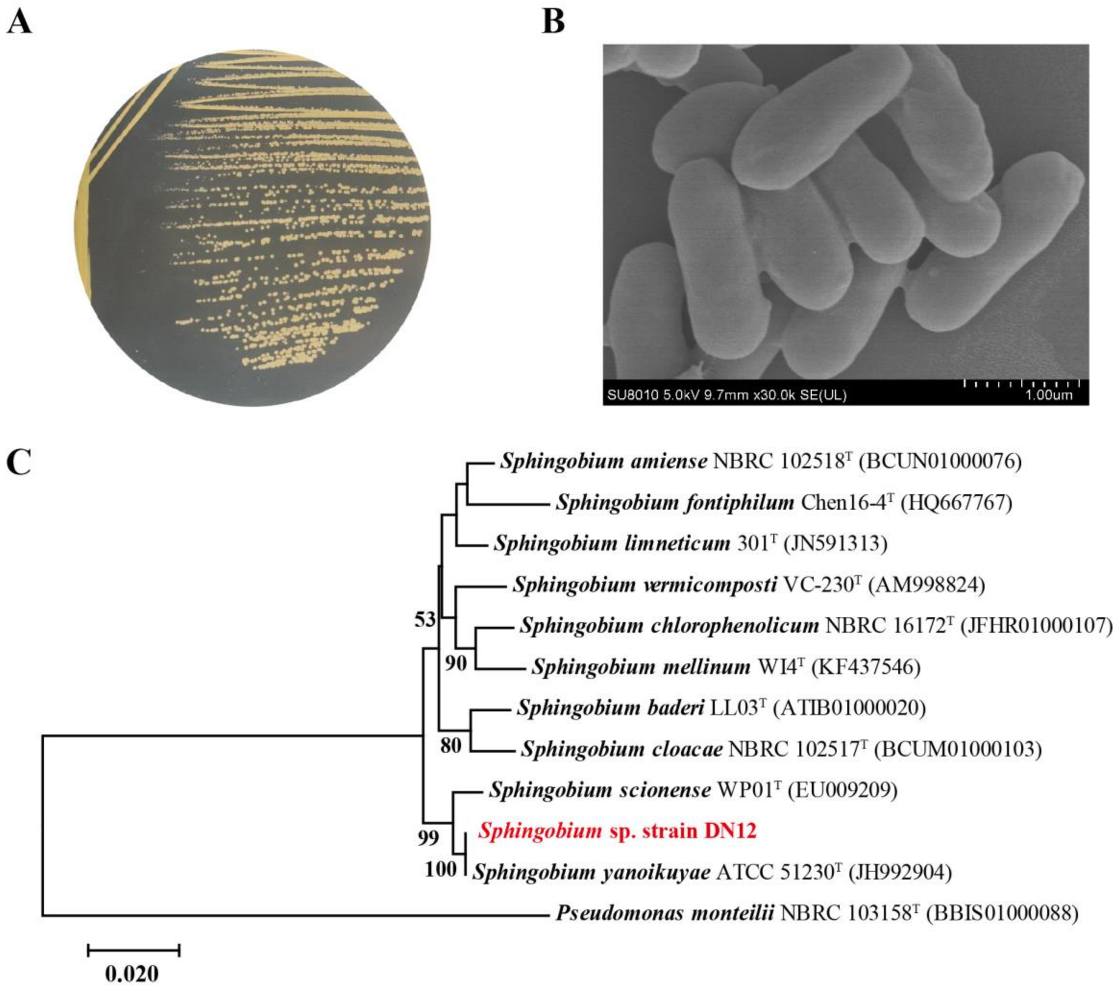

Fig. S1 Morphology and phylogenetic analysis of strain DN12. (A) Colony morphology of strain DN12 on an LB agar plate. (B) Scanning electron micrograph of strain DN12 (scale bar = 1.0 μm). (C) Phylogenetic tree constructed based on the Neighbor-Joining method using 16S rRNA gene sequences of strain DN12 and closely related species. Bootstrap values greater than 50% (based on 1000 replicates) are shown at the branch nodes. The scale bar represents 0.020 changes per nucleotide position.

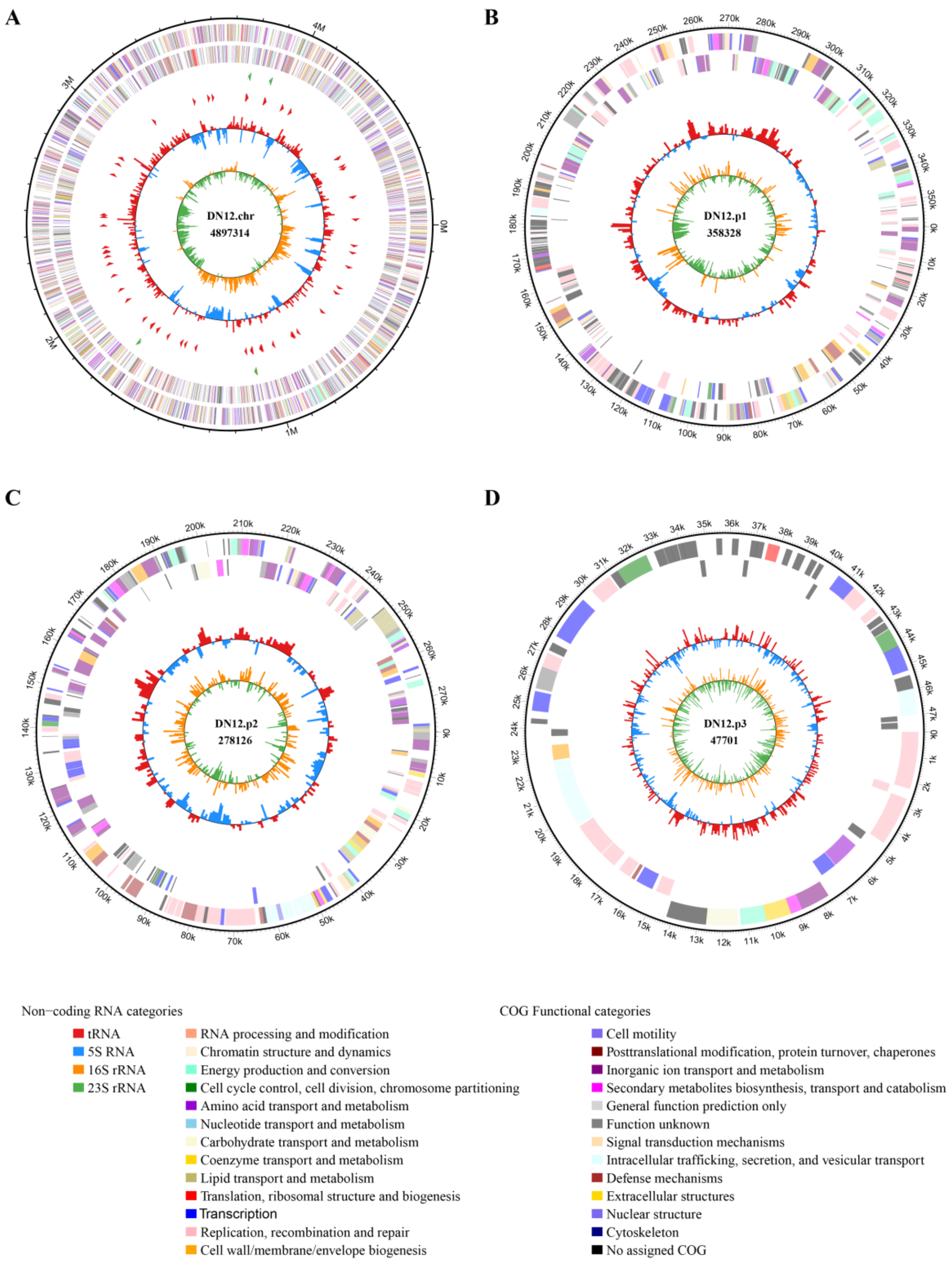

Fig. S2 Circular representation and genetic features of the chromosome (A), pDN-1 (B), pDN-2 (C), and pDN-3 (D) of strain DN12. From outside to center: genes on the forward strand (colored by COG categories).

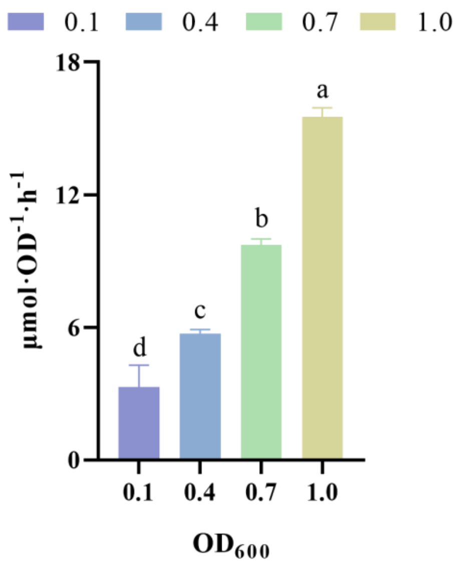

Fig. S3 Specific degradation activities of strain DN12 at different initial inoculum densities. Specific activities were calculated as μmol BPF degraded per OD_{600} per hour, based on the degradation of 0.10-0.16 mM BPF during the linear degradation phase. Bars represent the mean ± standard deviation from three biological replicates.

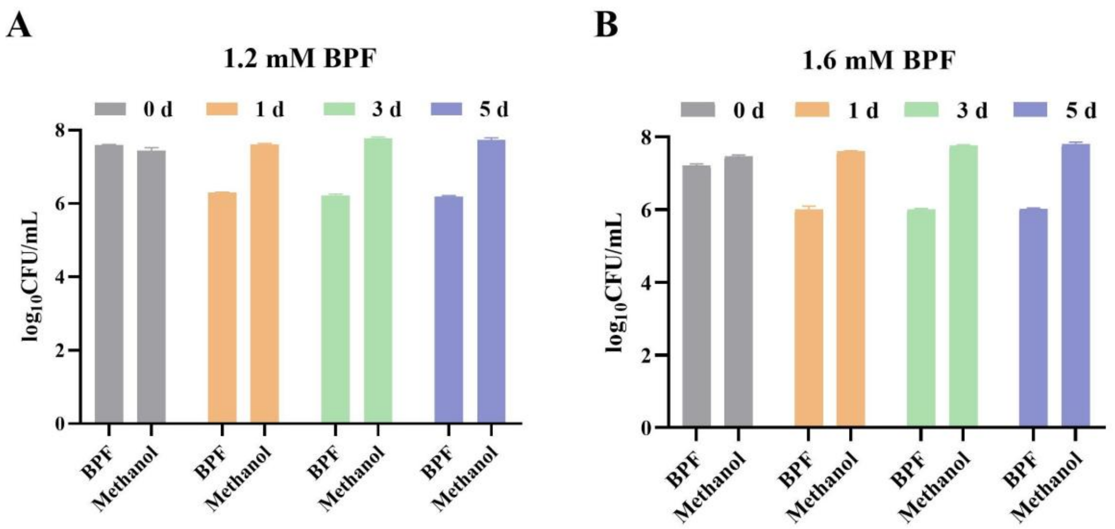

Fig. S4 CFU-counts of strain DN12 at 0, 1, 3, and 5 days after exposure to 1.2 mM (A) and 1.6 mM (B) BPF, compared with an equal volume of methanol. Cell numbers are expressed as $\log_{10}$(CFU/mL) and represent the mean ± standard deviation of three biological replicates.

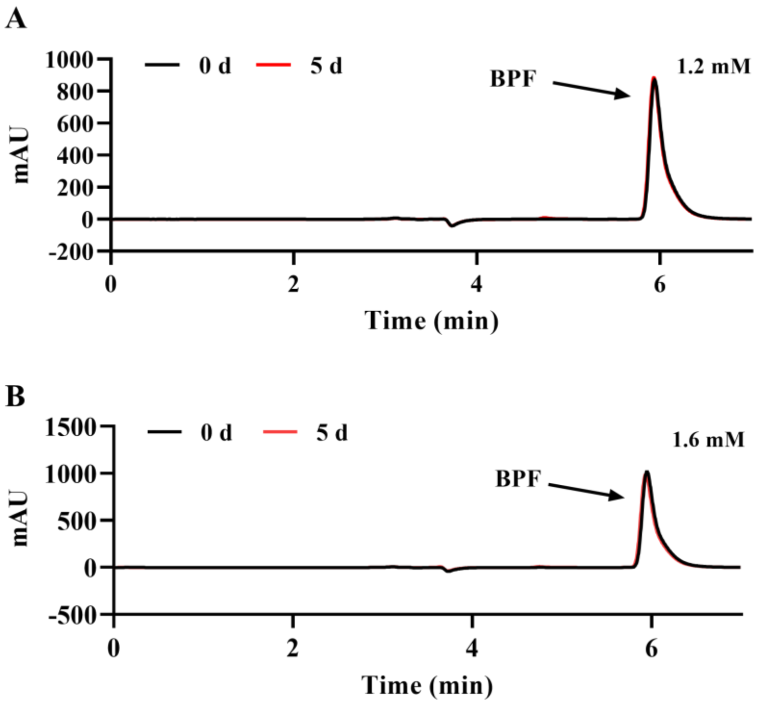

Fig. S5 HPLC analysis of BPF degradation by strain DN12 after 0 and 5 days with 1.2 mM (A) and 1.6 mM (B) BPF.

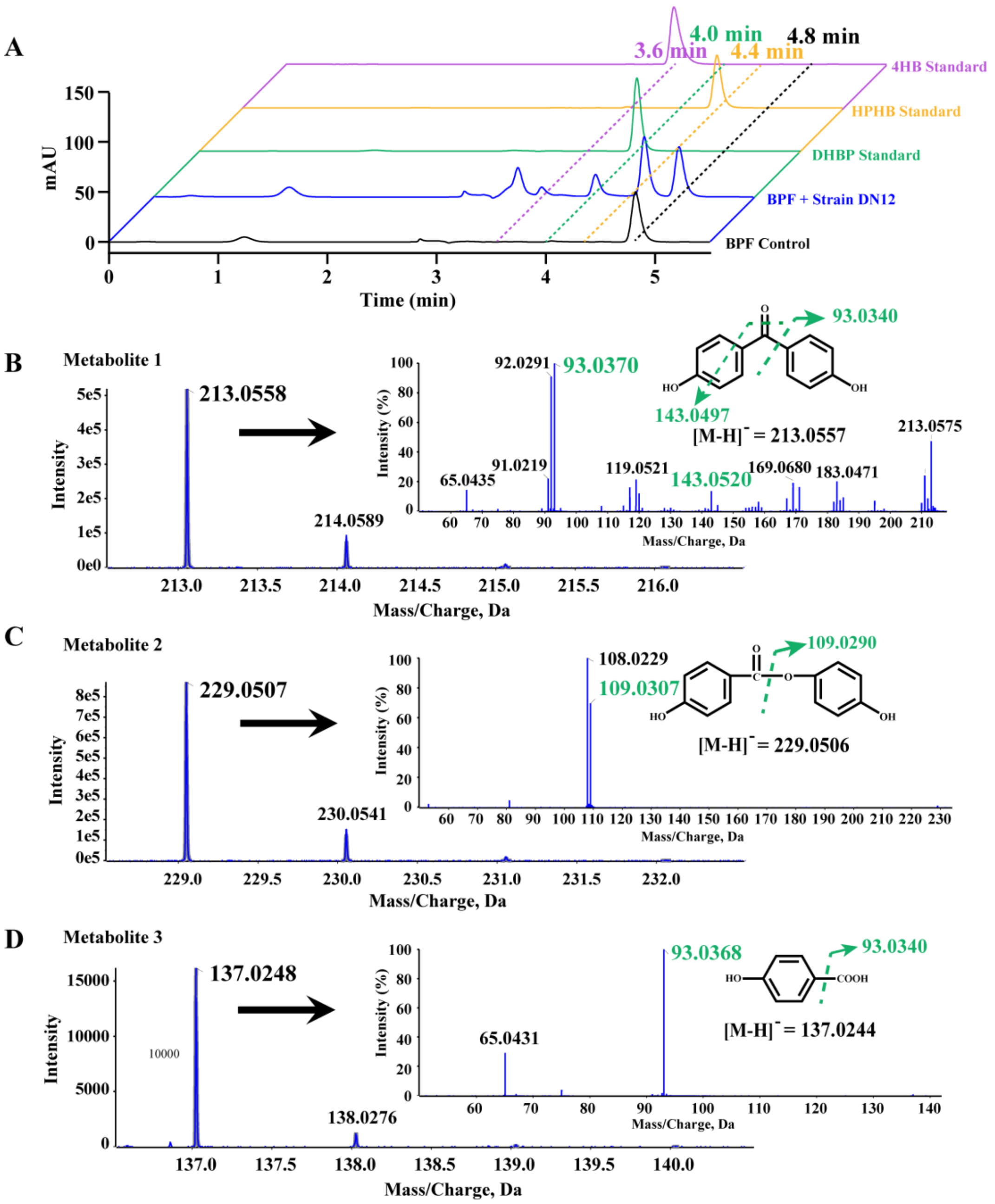

Fig. S6 Identification of three metabolites produced during BPF degradation by strain DN12. (A) HPLC analysis of BPF degradation by strain DN12. The black line represents the control (uninoculated strain DN12), the blue line represents the BPF degradation by strain DN12, while the green, yellow, and purple lines correspond to the standards of DHBP, HPHB, and 4HB, respectively. HRMS/MS analysis of the three metabolites: DHBP (B), HPHB (C), and 4HB (D), generated during BPF degradation by strain DN12.

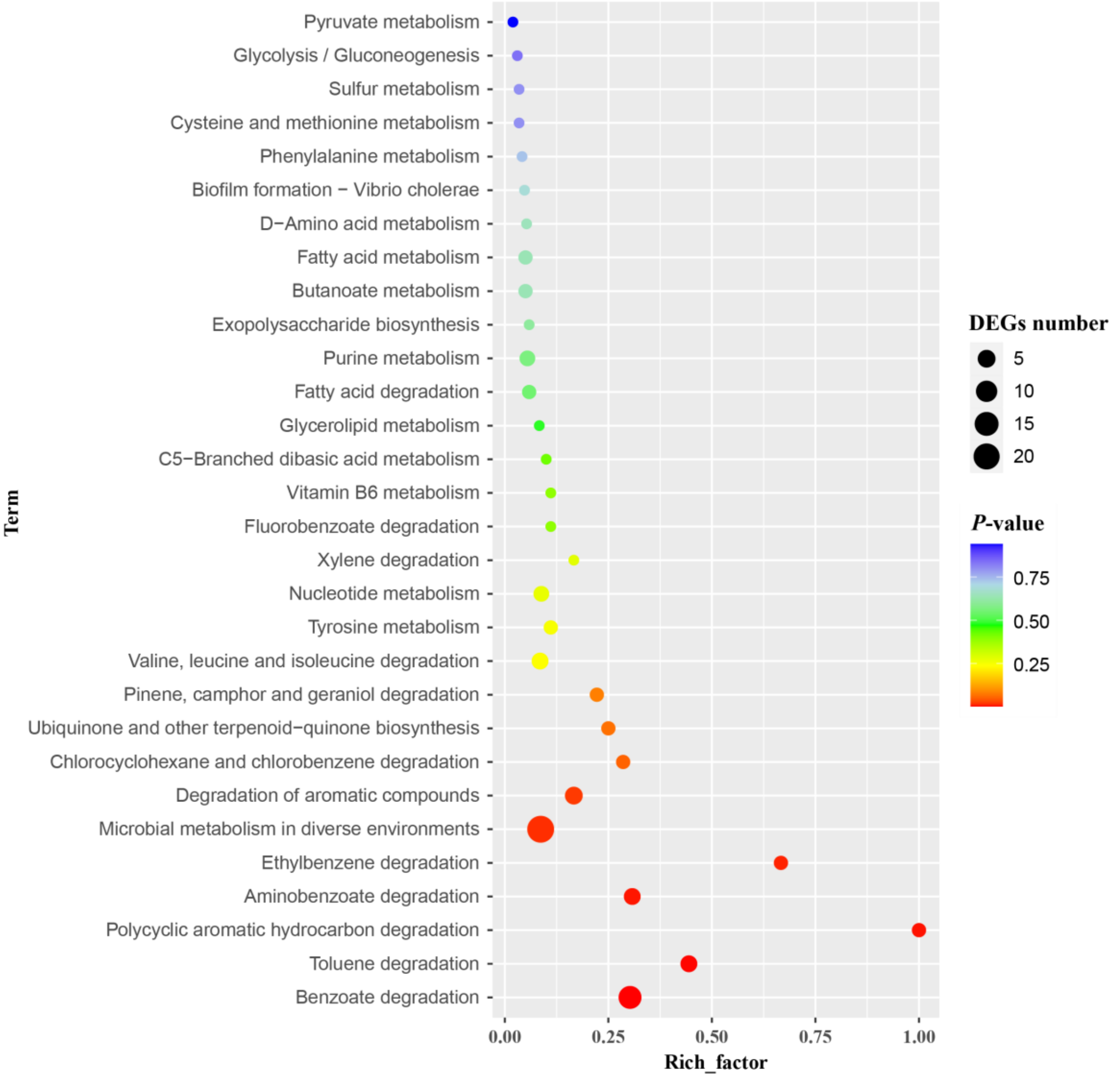

Fig. S7 Bubble graph of the Kyoto Encyclopedia of Genes and Genomes enrichment pathway analysis. Bubble color indicates *P* value, and bubble size represents the DEG numbers.

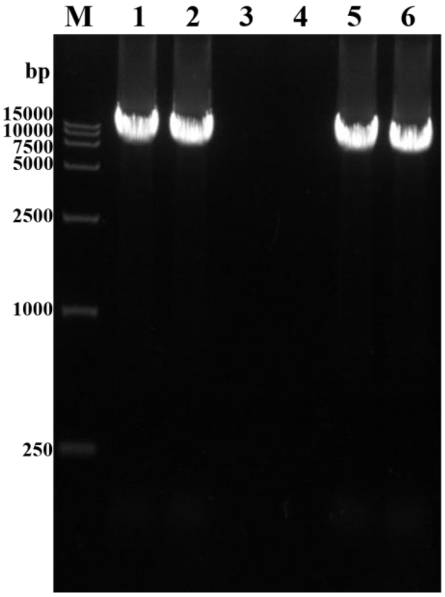

Fig. S8 Agarose gel electrophoresis analysis of PCR products for verification of the *bpf* cluster deletion and complementation. Lane M: DL15000 DNA marker; lane 1: PCR amplification of *bpf1* from the wild-type strain DN12; lane 2: PCR amplification of *bpf2* from the wild-type strain DN12; lane 3: PCR amplification of *bpf1* from the mutant strain DN12Δ*bpf*; lane 4: PCR amplification of *bpf2* from the mutant strain DN12Δ*bpf*; lane 5: PCR amplification of *bpf1* from the complemented strain DN12Δ*bpf* (pBBR-*bpf1*); lane 6: PCR amplification of *bpf2* from the complemented strain DN12Δ*bpf* (pBBR-*bpf2*).

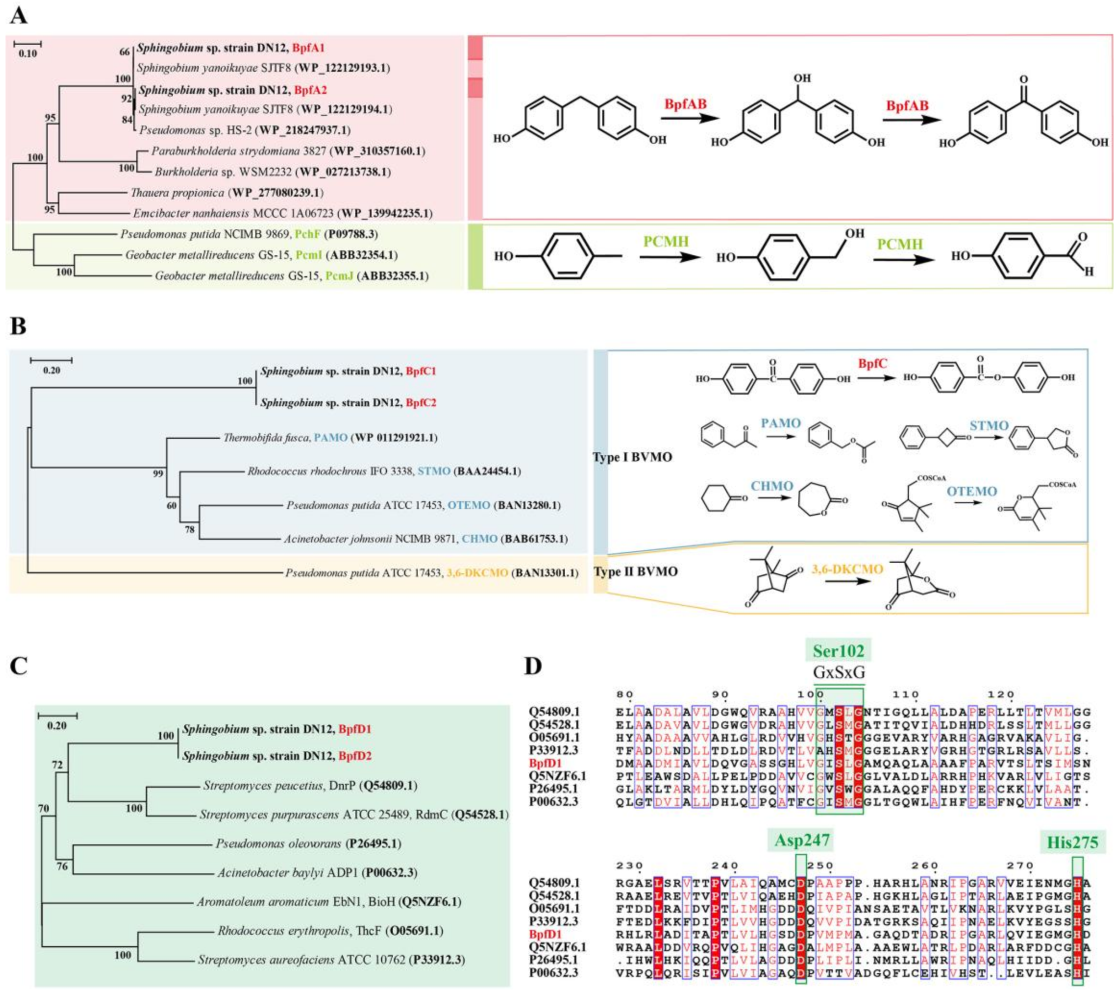

Fig. S9 (A) Phylogenetic analysis of BpfA showing its evolutionary relationship with highly homologous proteins in the NCBI public database, as well as the types of reactions catalyzed by BpfAB and proteins with similar functions reported. (B) Phylogenetic analysis of BpfC showing its relationship to homologous proteins in the SwissProt database, including the types of reactions catalyzed by BpfC and similar enzymes. (C) Phylogenetic analysis of BpfD and its relationship to homologous proteins in the SwissProt database. (D) Amino acid sequence alignment of BpfD with closely related homologs. Conserved domains are highlighted in red, and residues proposed to contribute to BpfD substrate specificity are boxed in green.

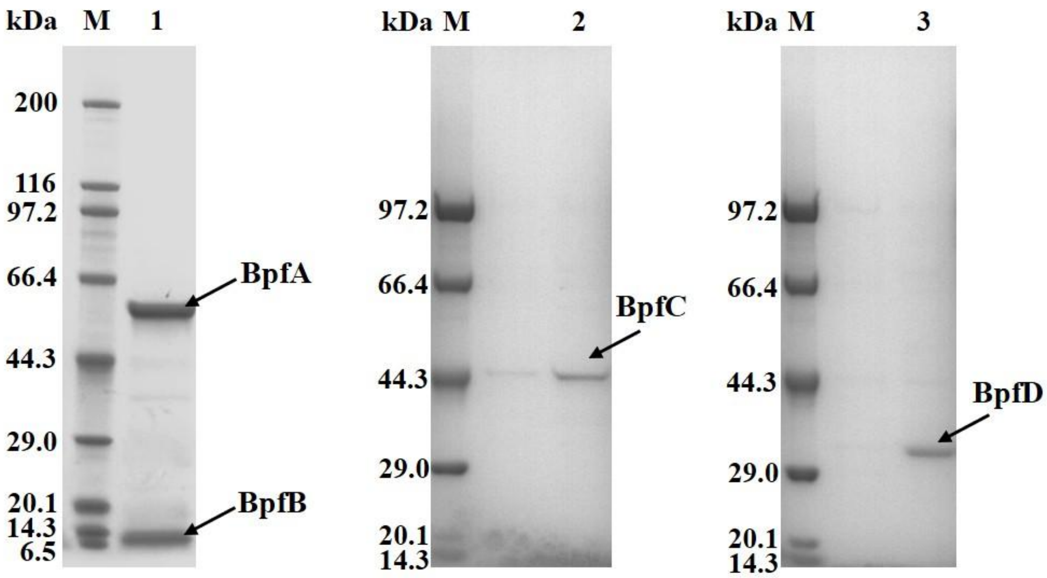

Fig. S10 SDS-PAGE analysis of purified BpfAB, BpfC and BpfD. Lane M, molecular weight markers; lane 1, purified BpfAB; lane 2, purified BpfC; lane 3, purified BpfD.

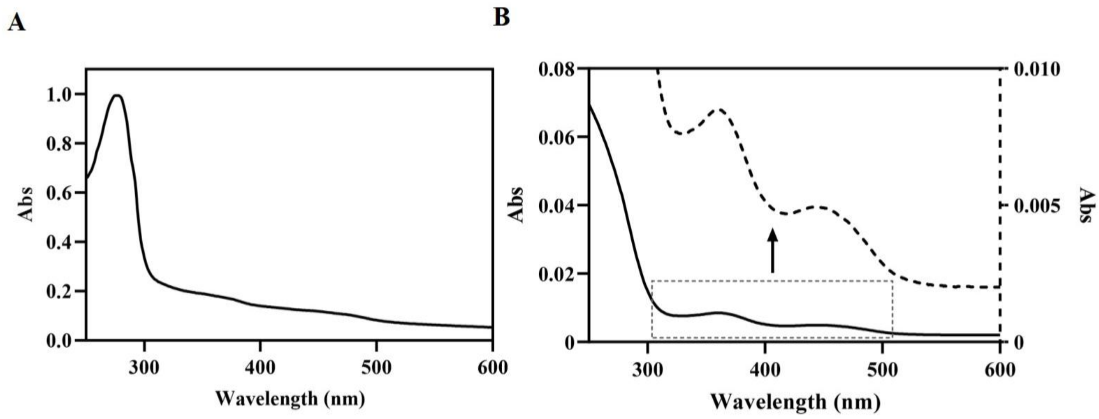

Fig. S11 (A) UV-vis absorption spectrum of purified BpfAB. The spectrum was normalized to the absorbance at 280 nm. (B) UV-vis absorption spectrum of FAD in BpfAB, highlighting characteristic FAD absorption peaks at ~375 nm and 450 nm.

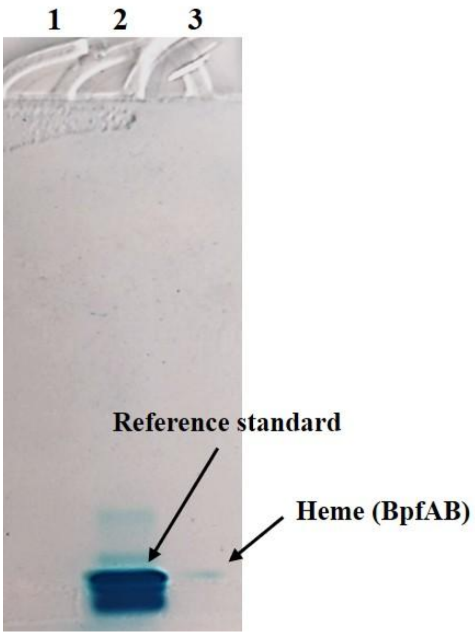

Fig. S12 Heme staining of purified proteins using the TMB assay. Lane 1, purified BpfA (negative control); lane 2, cytochrome *c* reference standard (positive control); lane 3, purified BpfAB complex.

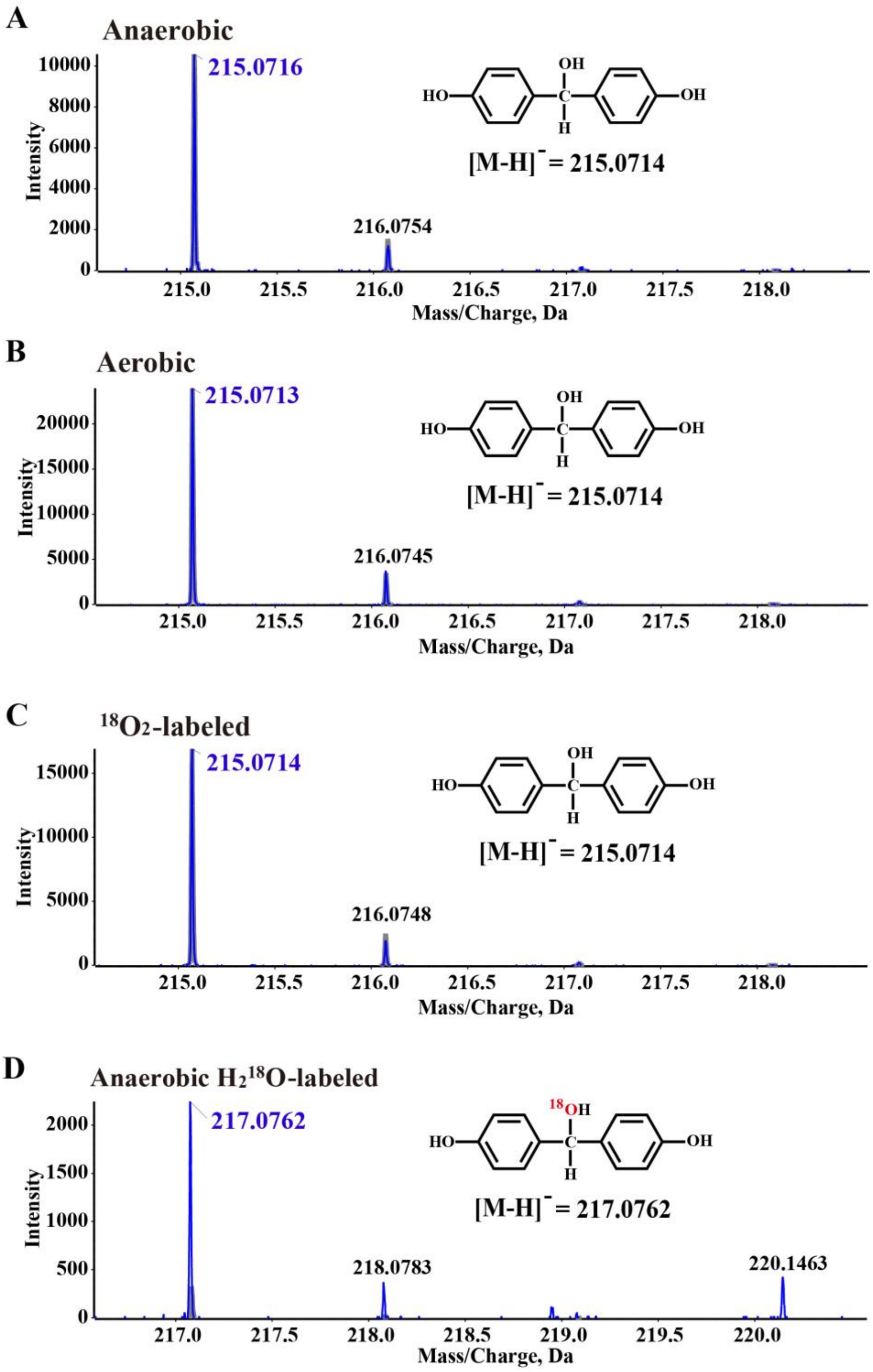

Fig. S13 HRMS analysis of bis(4-hydroxyphenyl)methanol, the product of BPF transformed by BpfAB under anaerobic (A), aerobic (B), $^{18}O_2$-labeled (C), and anaerobic $H_2{}^{18}O$-labeled (D) conditions.

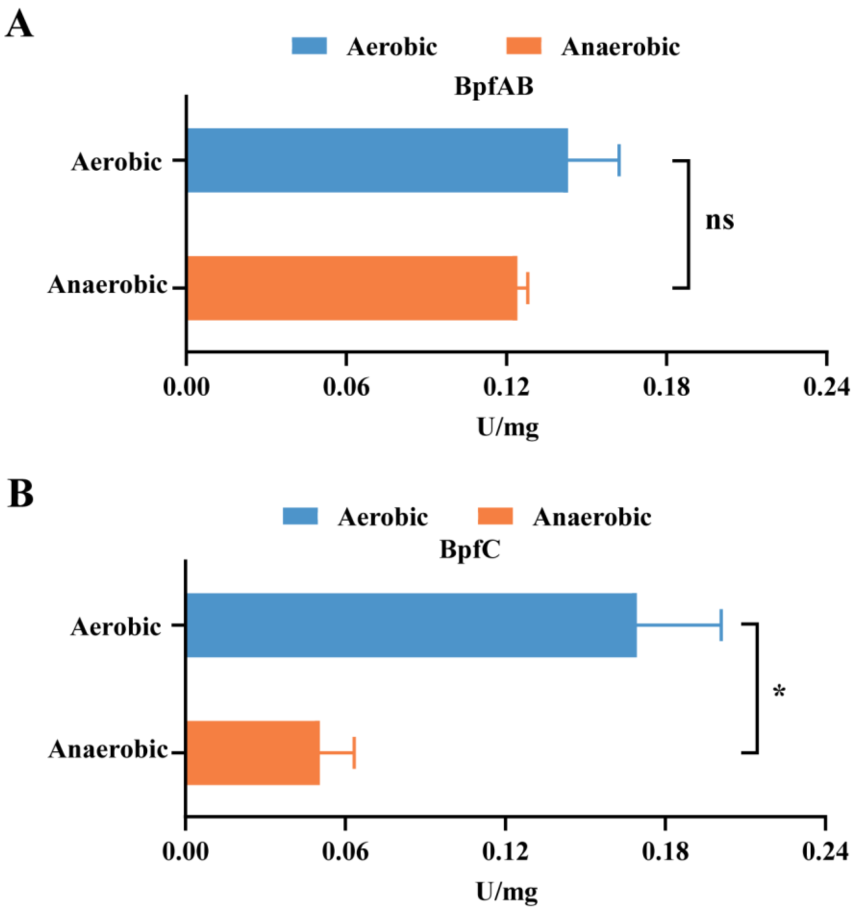

Fig. S14 (A) Specific activity of BpfAB toward BPF under anaerobic and aerobic conditions. (B) Specific activity of BpfC toward DHBP under anaerobic and aerobic conditions. "*" indicates a significant difference ($P < 0.05$), while "ns" indicates no significant difference ($P > 0.05$). Error bars represent the standard deviation of three biological replicates.

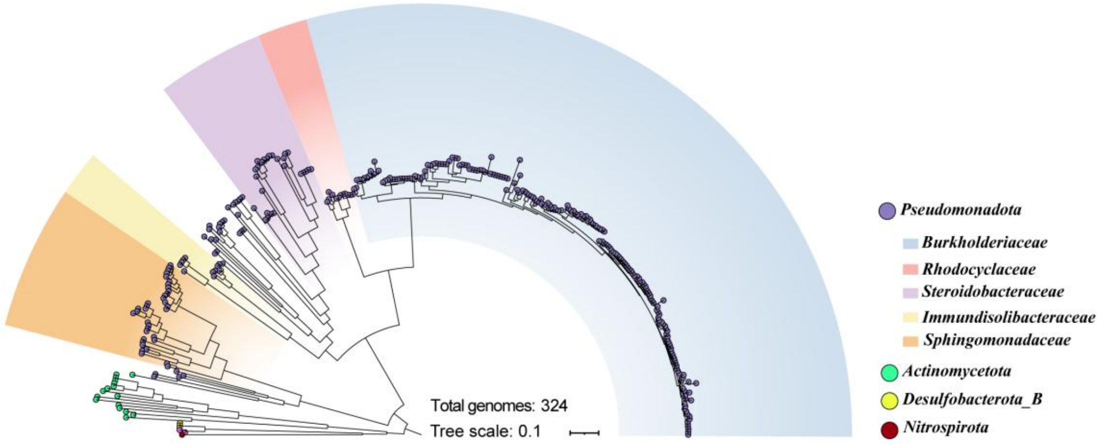

Fig. S15 Taxonomic distribution of 324 genomes encoding BpfA-like proteins. The phylogenetic bacterial genome tree was constructed based on concatenated alignment of 120 single-copy marker genes using GTDB-Tk.

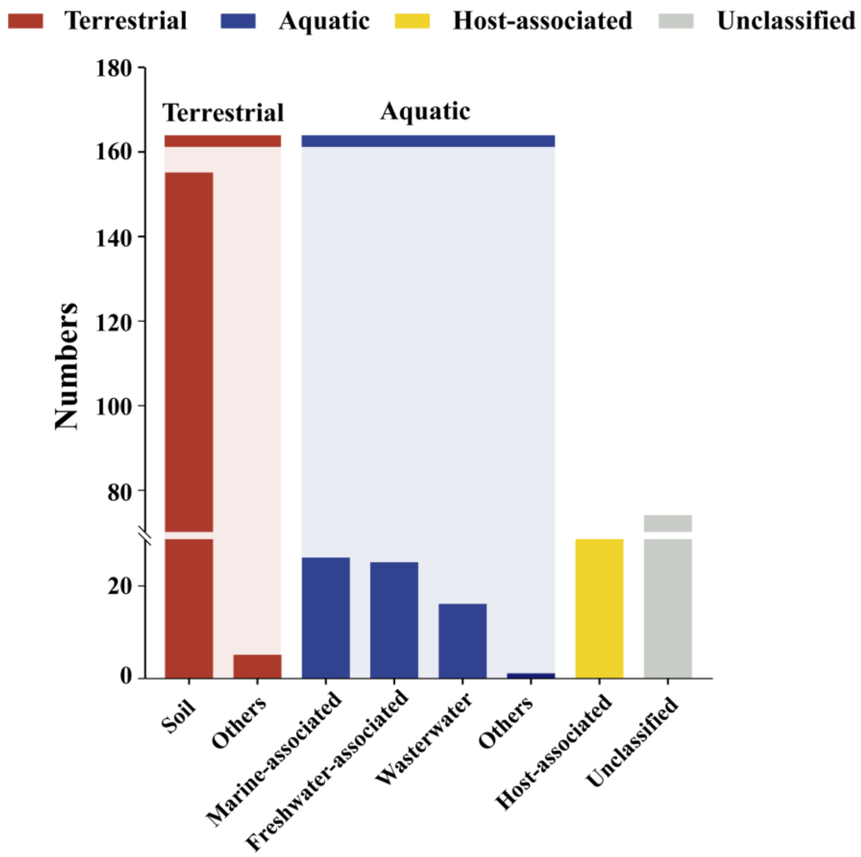

Fig. S16 Distribution of habitat types and their corresponding numbers among 325 genomes.

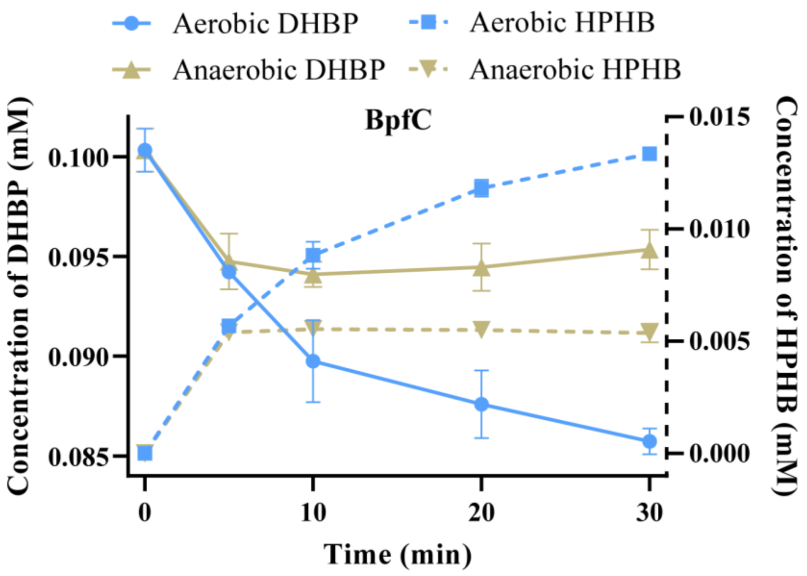

Fig. S17 Time-course of HPHB formation from DHBP catalyzed by BpfC under aerobic and anaerobic (N_2-purged) conditions. Data represent the mean ± standard deviation from three independent experiments.

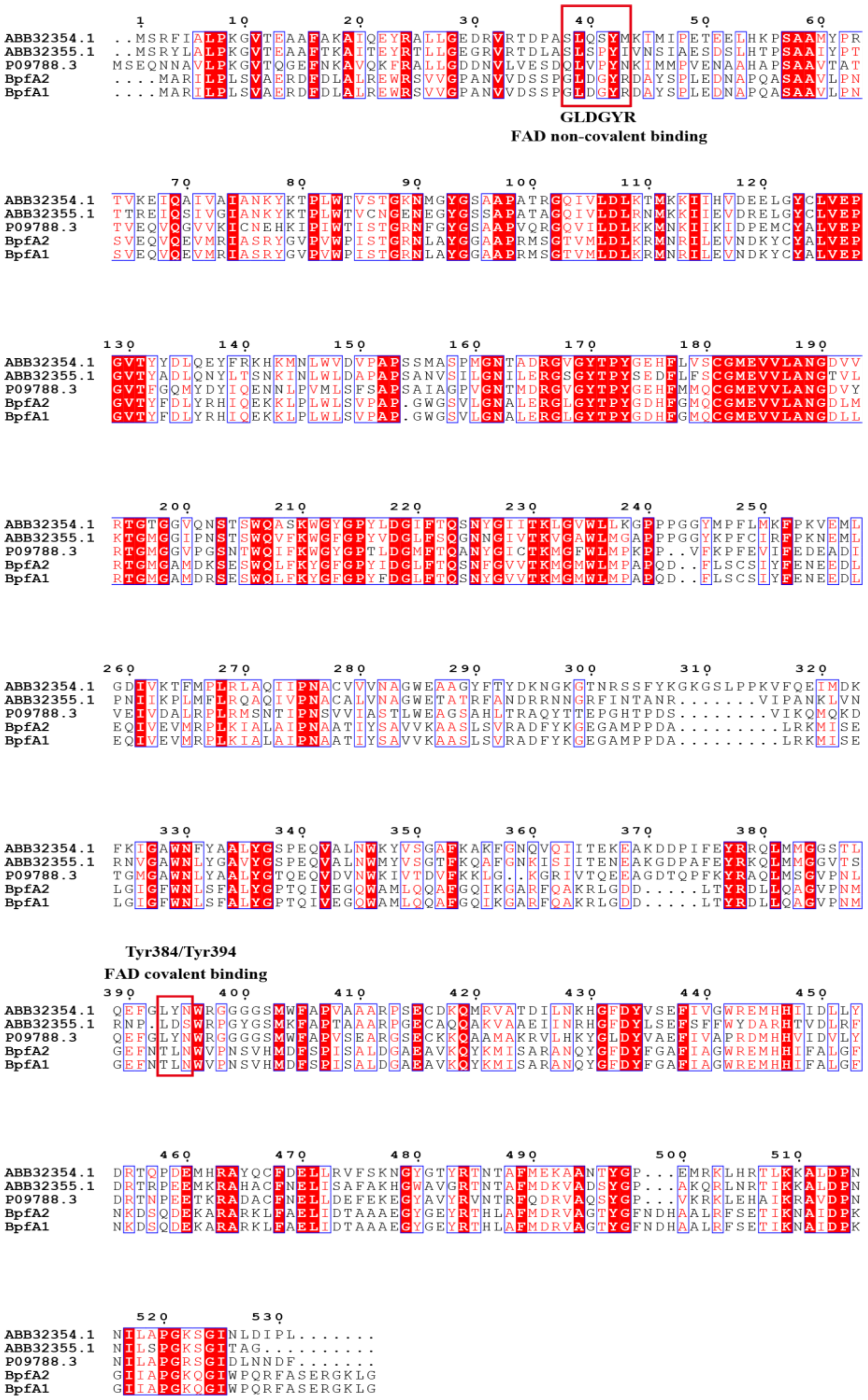

Fig. S18 Amino acid sequence alignment of BpfA with homologous proteins PchF (*Pseudomonas putida*, P09788.3), PcmI (*Geobacter metallireducens*, ABB32354.1), and PcmJ (*Geobacter metallireducens*, ABB32355.1). Conserved residues associated with covalent FAD attachment and non-covalent FAD binding motifs are highlighted with red boxes.

Table S1. Sensitivity of strain DN12 to antimicrobial agents.

Antibiotics	Results[a]	Antibiotics	Results[a]
Kanamycin	-	Streptomycin	+
Ampicillin	-	Gentamicin	-
Spectinomycin	-	Tetracycline	-
Chloramphenicol	-	Ofloxacin	-

[a] "-" indicates sensitivity of the strain to the antibiotic, while "+" signifies resistance.

Table S2. Genome information of the strain DN12.

Features	Base number (bp)	G+C content (%)	Gene number
Genome	5581469	63.23	5416
Chromosome	4897314	64.46	4689
Plasmid 1	358328	62.53	381
Plasmid 2	278126	62.68	284
Plasmid 3	47701	62.11	62

Table S3. Degradation of bisphenol analogs by strain DN12.

Bisphenol analogs	CAS number	Chemical structure formula	Degradation Capability [a]
BPA	80-05-7	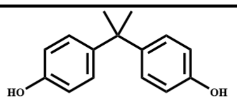	-
BPS	80-09-1	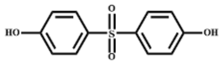	-
BPF	620-92-8	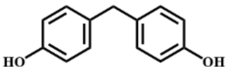	+
BPB	77-40-7	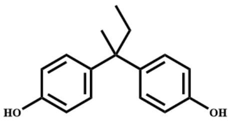	-
BPE	2081-08-5	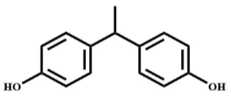	-
TDP	2664-63-3	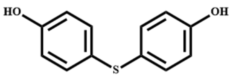	-
TBBPA	79-94-7	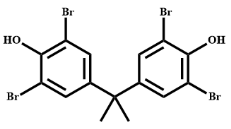	-
TBBPS	39635-79-5	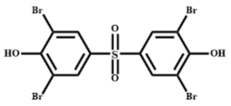	-

[a] The degradation efficiency of strain DN12 against various bisphenol analogs was analyzed using by HPLC after a 2-day incubation period. "+" indicates degradation; "-" signifies no degradation.

Table S4. Transcripts with up-regulation (log_2 fold change >2) in BPF-induced cells compared with those in uninduced cells.

orf (gene)	Product size [a]	log_2 fold change [b]	Database [c]	Proteins with highest identity, function, host	GenBank accession No.	Identity value
orf582	393	3.95	Swiss-Prot	*p*-hydroxybenzoate hydroxylase, PHBH, *Pseudomonas aeruginosa* PAO1	P20586	60%
orf583	256	2.15	Swiss-Prot	Uncharacterized Nudix hydrolase, NudL, *Cronobacter sakazakii* ATCC BAA-894	A7ZMT6	37%
orf622	203	3.12	Swiss-Prot	Epimerase family protein, YfcH, *Escherichia coli* K-12	P77775	43%
orf939	211	2.03	Swiss-Prot	Aldehyde oxidoreductase iron-sulfur-binding subunit, PaoA, *Escherichia coli* O157:H7	Q8X6I9	64%
orf1034	92	3.33	NR	Hypothetical protein, *Sphingobium* sp. YR657	SHM73423	98%
orf1658	92	3.33	NR	Hypothetical protein, *Sphingobium* sp. YR657	SHM73423	98%

orf1810	328	3.63	Swiss-Prot	Uncharacterized oxidoreductase, YccK, *Bacillus subtilis* strain 168	P46905	36%
orf2927	313	3.23	Swiss-Prot	4-carboxy-2-hydroxymuconate-6-semialdehyde dehydrogenas, *Sphingobium* sp. SYK-6	Q9KWL3	99%
orf2928	282	2.79	Swiss-Prot	Protocatechuate 4,5-dioxygenase beta chain, *Sphingobium* sp. SYK-6	P22636	69%
orf2929	134	2.82	Swiss-Prot	Protocatechuate 4,5-dioxygenase alpha chain, *Sphingobium* sp. SYK-6	P22635	70%
orf2930	340	3.25	Swiss-Prot	2-keto-4-carboxy-3-hexenedioate hydratase, *Sphingobium* sp. SYK-6	G2IQQ5	86%
orf2931	298	2.01	PDB	NAD-binding phosphogluconate dehydrogenase-like protein, *Alicyclobacillus acidocaldarius* subsp. *acidocaldarius* DSM 446	3QSG_A	34%
orf2934	354	2.51	Swiss-Prot	(4E)-oxalomesaconate Delta-isomerase, *Novosphingobium* sp. KA1	Q0KJL4	82%

orf2935	295	3.14	Swiss-Prot	2-pyrone-4,6-dicarboxylate hydrolase, Sphingobium sp. SYK-6	O87170	87%
orf3128	126	2.85	NR	OsmC family protein, *Sphingobium yanoikuyae*	WP_125989724	98%
orf3129	298	3.94	Swiss-Prot	Pirin-like protein CC_3178, *Caulobacter vibrioides* CB15	P58114	52%
orf3130	106	3.49	NR	Hypothetical protein, *Sphingobium yanoikuyae*	WP_374551500	98%
orf3131	232	3.49	Swiss-Prot	Pirin-like protein CC_1473, Caulobacter vibrioides CB15	P58113	72%
orf3586	92	3.33	NR	Hypothetical protein, *Sphingobium* sp. YR657	SHM73423	98%
orf3687	337	3.33	Swiss-Prot	Uncharacterized oxidoreductase, YccK, *Bacillus subtilis* strain 168	P46905	36%
orf5013	838	3.91	Swiss-Prot	Vitamin B12 transporter BtuB, *Vibrio vulnificus* CMCP6	Q8DD41	32%

orf5014	248	2.94	Swiss-Prot	3-alpha-(or 20-beta)-hydroxysteroid Dehydrogenase, *Mycobacterium tuberculosis variant bovis* AF2122/97	P69166	36%
orf5015 (bpfC1)	402	3.19	Swiss-Prot	FAD-dependent monooxygenase, *Stachybotrys chartarum* IBT 7711	A0A084B9Z5	44%
orf5016 (bpfD1)	301	3.41	Swiss-Prot	Aclacinomycin methylesterase, RdmC, *Streptomyces purpurascens*	Q54528	29%
orf5017	189	2.90	NR	Hypothetical protein, *Sphingobium yanoikuyae*	WP_122129108	100%
orf5018 (bpfB1)	141	3.16	NR	Cytochrome c, *Sphingobium yanoikuyae*	WP_122129109	100%
orf5019 (bpfA1)	522	3.21	Swiss-Prot	*P*-cresol methylhydroxylase, PCMH, *Pseudomonas putida*	P09788	43%
orf5022 (bpfA2)	522	3.35	Swiss-Prot	*P*-cresol methylhydroxylase, PCMH, *Pseudomonas putida*	P09788	43%
orf5023	141	3.16	NR	Cytochrome c, *Sphingobium yanoikuyae*	WP_122129109	100%

(bpfB2)						
orf5024	189	2.90	NR	Hypothetical protein, *Sphingobium yanoikuyae*	WP_122129108	100%
orf5025 (bpfD2)	301	3.41	Swiss-Prot	Aclacinomycin methylesterase, RdmC, *Streptomyces purpurascens*	Q54528	29%
orf5026 (bpfC2)	402	3.15	Swiss-Prot	FAD-dependent monooxygenase, *Stachybotrys chartarum* IBT 7711	A0A084B9Z5	44%
orf5027	248	2.35	Swiss-Prot	Pyridoxal 4-dehydrogenase, *Mesorhizobium japonicum* MAFF 303099	Q988B7	39%
orf5031	496	2.30	Swiss-Prot	4-hydroxybenzaldehyde dehydrogenase (NADP(+)), *Pseudomonas putida*	Q59702	59%
orf5044	377	2.32	Swiss-Prot	Beta-ketoadipyl-CoA thiolase, *Pseudomonas aeruginosa* PAO1	Q9I6R0	73%
orf5045	225	2.74	Swiss-Prot	Succinyl-CoA:3-ketoacid coenzyme A transferase subunit B, *Xanthomonas campestris pv. campestris str.* ATCC 33913	P0C7I8	67%

orf5046	236	2.74	Swiss-Prot	Succinyl-CoA:3-ketoacid coenzyme A transferase subunit A, *Xanthomonas campestris pv. campestris str.* B100	B0RVK4	68%
orf5048	170	3.76	PDB	Hydroquinone dioxygenase small subunit, *Sphingomonas* sp. TTNP3	5M21_A	100%
orf5049	339	4.66	PDB	Hydroquinone dioxygenase large subunit, *Sphingomonas* sp. TTNP3	5M21_B	100%
orf5050	328	4.40	NR	hypothetical protein EBF16_01375, *Sphingobium yanoikuyae*	AYO75670	100%
orf5051	498	4.08	Swiss-Prot	Putative aldehyde dehydrogenase, DhaS, *Bacillus subtilis* strain 168	O34660	44%
orf5052	352	3.21	Swiss-Prot	Maleylacetate reductase, *Pseudomonas* sp. P51	P27101	55%
orf5053	275	2.43	Swiss-Prot	Hydroxyquinol 1,2-dioxygenase, *Rhizobium* sp. MTP-10005	A1IIX3	53%

orf5055	193	4.61	Swiss-Prot	UPF0312 protein VCM66_A0498, *Vibrio cholerae* M66-2	C3LVF6	30%
orf5056	185	4.64	Swiss-Prot	Cytochrome b561 homolog 2, *Escherichia coli* K-12	P75925	32%
orf5057	203	4.72	Swiss-Prot	UPF0312 protein PSPPH_0448, *Pseudomonas savastanoi pv. phaseolicola* 1448A	Q48PB8	36%
orf5058	182	4.38	Swiss-Prot	NAD(P)H dehydrogenase (quinone), *Azorhizobium caulinodans* ORS 571	A8HRS7	83%
orf5060	261	3.24	Swiss-Prot	Pirin-like protein CC_3178, *Caulobacter vibrioides* CB15	P58114	48%

[a] Number of amino acids.

[b] Average ratio of BPF-induced to BPF-uninduced cells from three biological replicates for RNA-seq (mRNA); RPKM with log_2fold change >2 (BPF-induced/uninduced) are considered significantly more abundant in the presence of BPF (P <0.05).

[c] NR, NCBI Non-redundant protein sequences database; PDB, Protein Data Bank proteins.
